# Prestress Monitoring of a Steel Strand in an Anchorage Connection Using Piezoceramic Transducers and Time Reversal Method

**DOI:** 10.3390/s18114018

**Published:** 2018-11-18

**Authors:** Xiaoyu Zhang, Liuyu Zhang, Laijun Liu, Linsheng Huo

**Affiliations:** 1 Engineering Research Center for Large Highway Structure Safety of the Ministry of Education, Chang’an University, Xi’an 710064, China; xiaozhangyu2018@163.com (X.Z.); liulj@chd.edu.cn (L.L.); 2 State Key Laboratory of Coastal and Offshore Engineering, Dalian University of Technology, Dalian 116024, China

**Keywords:** piezoceramics, Lead Zirconate Titanate (PZT), prestress monitoring, steel strand, wedge anchorage system, time reversal method

## Abstract

Steel strands are widely used in cable stay or suspension bridges. The safety and stability of steel strands are important issues during their operation period. Steel strand is subjected to various types of prestress loss which loosens the wedge anchorage system, negatively impacting the stability of the structure and even leading to severe accidents. In this paper, the authors propose a time reversal (TR) method to monitor the looseness status of the wedge anchorage system by using stress wave based active sensing. As a commonly used piezoceramic material, Lead Zirconate Titanate (PZT) with a strong piezoelectric effect is employed. In the proposed active sensing approach, PZT patches are used as sensors and actuators to monitor the steel strand looseness status. One PZT patch is bonded to the steel strand, one PZT patch is bonded to the wedges, and another PZT patch is bonded to the barrel. There are three different interfaces of the wedge anchorage system to monitor the steel strand looseness status. In the first method, the PZT patch on the steel strand is used as an actuator to generate a stress wave and the PZT patch on the wedge is used as a sensor to detect the propagated waves through the wedge anchorage system. In the second method, the PZT patch on the steel strand is used as an actuator to generate a stress wave and the PZT patch on the barrel is used as a sensor to detect the propagated waves through the wedge anchorage system. In the third method, the PZT patch on the wedges is used as an actuator to generate a stress wave and the PZT patches on the barrel is used as a sensor to detect the propagated waves through the wedge anchorage system, of which the looseness will directly impact the stress wave propagation. The TR method is utilized to analyze the transmitted signal between PZT patches through the wedge anchorage system. Compared with the peak values of the TR focused signals, it can be found that the peak value increases as the wedge anchorage system tightness increases. Therefore, the peak value of the TR focused signal can be used to monitor the tightness of the steel strand. In addition, the experimental results demonstrated the time reversal method’s reliability, sensitivity and anti-noise property.

## 1. Introduction

The long-span prestressed concrete bridge has many advantages, such as large span capacity, strong seismic capacity, convenient construction and little maintenance work. It is part of the fastest developing and widely used structural forms in the modern bridge engineering field [1]. The steel strand is the main component in the prestressed structure and its stress state directly affects the applicability and safety of the prestressed structure [2,3]. A series of failure of prestressed concrete bridges resulted in collapses all over the world, and most of these accidents were caused by the failure of prestressed steel strands. When the prestressed steel strands of the long-span prestressed concrete girder bridge lose the effective value, this may result in excessive deflection of the girder, finally affecting the driving comfort [4]. Moreover, the failure of the longitudinal prestress steel strand in the bottom of the positive bending moment is the main reason for the floor concrete cracking [5].

In fact, the effective prestress is time-dependent. Measured results show that the long-term loss of prestress could reach 16% of the effective prestress after the completion of long-span prestressed concrete girder bridge in eight years [6]. The loss of prestress is related to construction technology, material properties and environmental factors [7]. Except for the loss of prestress due to creep and shrinkage of concrete, friction between prestressed tendons and prestressed pipe, deformation of the anchorage, retraction of steel strand and compression of joints and other aspects of the bridge have great influence on the effective prestress. Therefore, steel strand stress monitoring is significantly important. Currently, commonly used sensors for the stress detection of steel strands include electric resistance strain gauge, vibrating string extensometer, fiber grating strain gauge and steel strand strain transducer. Traditional resistance strain gauges are prone to corrosion [8]. The prestressed concrete bridge with its boundary conditions, stress amplitude and workspace are obviously different from the cable and suspender, so the vibration frequency method is not suitable for concrete bridges. Because fiber grating strain gauge is quite sensitive to temperature, it needs temperature compensation through a reference sensor [9,10]. Ultrasonic guided wave detection in cable and anchorage systems must be strictly demarcated, however the steel strand itself has clearance and may have original defects at the beginning, so the results are not ideal [11,12]. Due to the complexly ferromagnetic environment around the steel strand, the anti-ferromagnetic ability should be determined [13,14]. Prestressing force gauge prestressed at the external anchor head can effectively monitor the change of working stress of prestressed steel strand. However, due to the cost and relatively large size, the number of monitoring points are limited in the actual application process and the monitoring period is difficult to ensure adequate working hours.

In recent years, structural health monitoring (SHM) [15,16,17] that is capable of real time monitoring for the purposes of detecting structural damages and issuing early warnings [18,19,20,21] has received increasing attention in many different fields, including civil engineering [22,23,24,25]. In SHM, Lead Zirconate Titanate (PZT), a type of piezoelectric material, with the unique characteristic of both actuating and sensing capacities are commonly used as transducers to generate and detect stress waves, enabling stress-wave based monitoring methods [26,27,28], including the active sensing approach [29,30]. In addition, PZT transducers have a wide bandwidth and can be used to detect impacts [31,32,33,34] and explosions [35]. The time reversal method, which has the special property of spatial and temporal focusing [36,37], has also attracted wide attention in the SHM community in recent years [38,39,40,41,42]. Recent experimental studies indicated that the signal measurements of a series of surface-mounted PZT patches arrayed in specific locations of the examined member improve the damage assessment procedure providing better correlation between the local damage level and the values of the used damage index [43,44]. Further, relative works highlighted that the implementation of a network of PZT transducers provides more accurate results concerning the evaluation of damages in concrete structural members [45,46]. Especially for the shear-critical concrete members, most are brittle structures which may exhibit brittle sudden diagonal cracking. Transducers can obtain real-time detection data and get satisfactory detection results with the damage index values calculated by the network. In addition, the damage index values may be deterministic indicators before the structure occurred damage [47,48].

Wang et al. [49] employed a pair of surface-bonded two PZT patches across the interface of a bolted joint to monitor the pre-load on a bolt via the active sensing method. In particular, the energy index based on the active sensing method can effectively monitor the tightness of the bolt. Ji et al. [50] developed a time reversal enabled pulse-position modulation method to effectively solve the multipath propagation problem of stress-wave based communication in concrete structures. Watkins et al. [51] improved a lamb-wave based time reversal method for composite structure diagnosis and the results showed that the magnitude of the damage index has a direct relationship with the severity of the damage along the lamb wave path. Jun et al. [52] introduced a new digital image based damage detection method that is based on the arrival time via wave packet analysis. Vigoureux et al. [53] investigated the source identification problem using the time reversal method to detect the origin of the vibrations on the surface of the structure. Ciampa et al. [54] presented the inverse-time-reversal method to waveforms stored in a database. Using the advantages of multiple scattering, mode conversion and boundary reflection, focusing on high resolution sources is achieved. Ciampa et al. [55] proposed the time reversal method to monitor the effectiveness of the reinforced composite material in real time. By using the strengths of the diffusion wave field, the image of the source position is obtained through the virtual time reversal process and the position of the surface defect of the composite structure can be detected. Van Damme et al. [56] introduced a combination of time reversal acoustic and nonlinear elastic wave spectroscopy to monitor the surface crack of steel structures. The nonlinear elastic property of the steel crack interface is assigned to the sample surface, which can locate the location of the fracture surface on the industrial sample surface. Zhang et al. [57] applied the method of time reversal to monitor the structural safety of a scaffold. The monitoring results showed that the time reversal method could accurately monitor the tightness of the scaffold. There is a close correlation between the peak value of the focus reversal and the tightness of the scaffold structure.

It is imperative to develop a long-term, non-destructive and stable monitoring method for the health assessment of steel strands under diverse operating conditions. The piezoelectric ceramic transducer is a new type of stress monitoring method with the advantage of non-destructive, long-term and stable stress monitoring, and adapting to the construction status of prestressed steel strands is a good way to substitute the traditional monitoring means. At present, the stress monitoring experiment of the steel strand based on the piezoelectric ceramic sensor has not been reported. In this study, the time reversal method based on piezoceramic was used for active monitoring of the wedge anchorage system. Three piezoceramic patches were respectively pasted on the three different parts that were connected by the wedge anchorage system. Three piezoceramic pieces were respectively pasted on the three different positions that were connected by the wedge anchorage system, and three different contact surfaces were formed to monitor the anchoring tightness of the wedge anchorage system. In the proposed approach, one piezoceramic patch was used as an actuator to generate an elastic stress wave and another one was used to detect the wave that propagates through the wedge anchorage connection. One was the contact surface that was composed of the steel strands and wedges; the piezoceramic pasted to the surface of the wedges served as an actuator, and the piezoceramic pasted on the steel strands served as a sensor. One was the contact surface that was composed of the steel strands and barrel; the piezoceramic pasted to the surface of the steel strands served as an actuator, and the piezoceramic pasted on the barrel served as a sensor. One was a contact surface that was composed of a wedge and barrel; a piezoceramic pasted to the wedges served as an actuator, and a piezoceramic pasted on the barrel served as a sensor. The received energy depended on the contact area that was controlled by the tension of the steel strands which were controlled by different levels of digital jack. By analyzing the received signal based on time reversal, the loading status of the wedge anchorage can be monitored. To verify the effectiveness of the proposed method, a wedge anchorage system model was invoked as the test object. Experimental results clearly demonstrate that the proposed time reversal technique can monitor the loading status of the wedge anchorage system with excellent repeatability and strong anti-disturbance ability.

## 2. Principle for the Detection of Steel Strand Tightness Based on Time Reversal

### 2.1. Wedge Anchorage System Structure

**Figure 1:**
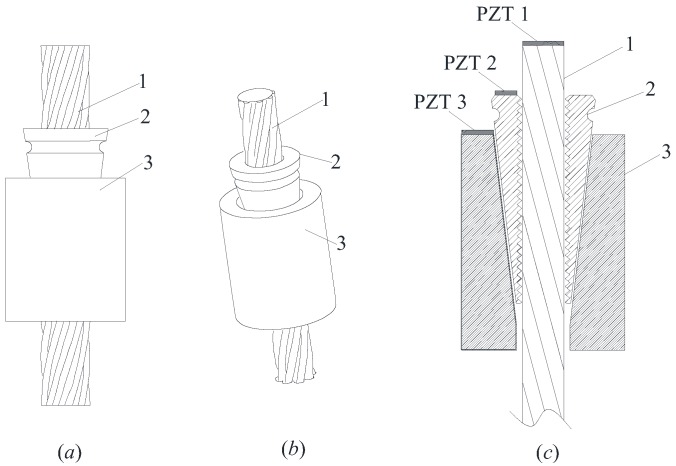
The examined wedge anchorage system and the installation of PZT (Lead Zirconate Titanate) transducers (**a**) 2D view (**b**) 3D view (**c**) cross-sectional view. 1: steel strand; 2: wedge; 3: barrel.

A wedge anchorage system commonly used in bridge construction is shown in Figure 1. Three components of the anchorage system are included: the steel strand, the wedge and the barrel.

### 2.2. Active Sensing Monitoring Principle Using Piezoelectric Transducers

**Figure 2:**
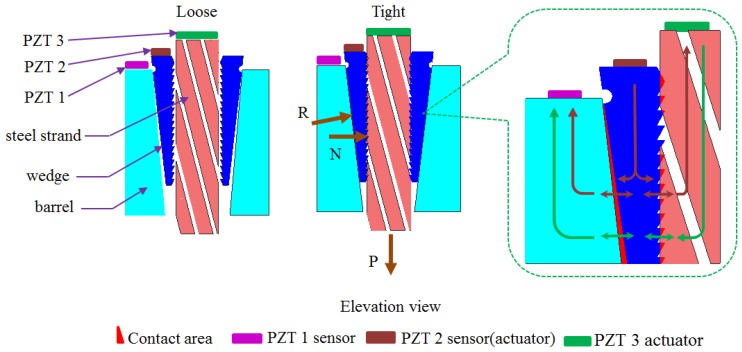
Microscopic view and energy transmission across contact interfaces for the wedge anchorage system.

As shown in Figure 2, PZT patches are surface-bonded on the end surfaces of the streel strand, the wedge and the barrel. In the literature, PZT patches, which are low cost, can be easily integrated with a variety of structures through surface bonding and have been widely used for vibration detection [58,59], vibration control [60,61,62,63,64], shape control [65], energy harvesting [66,67,68], stress wave generation [69], damage detection [70,71] and structural health monitoring [72,73]. In this research, PZT patches enable the stress wave based active sensing method to monitor the prestress on the steel strand.

As shown in Figure 2, owing to the tensile force P on the steel strand, the distributed pressure reaction R is formed on the outer side of the barrel. The horizontal force of R is the pressure N, which is the clamping force between the barrel and the steel strand acting on the steel strand. With the increase of the axial tension P of the steel strand, the inner hole of the wedges has teeth to bite the prestressed steel strand which drives the clip into the taper hole of the barrel. According to the wedge theory, the tighter the wedge, the greater the pressure N applied to the steel strand to provide enough clamping force to ensure the occlusive area between the steel strand and wedges is increasing. Meanwhile, distributed pressure reaction R formed on the outer side of the wedges increases continuously, which makes the inner contact surface of the barrel and the outer contact surface of the wedges extrude each other. When two rough elastic surfaces are extruded, the contact area is about proportional to the normal force. Therefore, with the contact area between the steel strand and wedges, the wedges and barrel will gradually increase once the strand is tensioned in the wedge anchorage system.

As shown in Figure 2, PZT 1, PZT 2 and PZT 3 are attached to the top of the steel strand, wedges and barrel, respectively. When PZT 2 is used as an actuator to generate a stress wave, the generated signal is transmitted to PZT 3 through the contact surface between the wedge and barrel. Meanwhile, the signal generated by PZT 2 can be transmitted to PZT 1 through the contact surface between the wedge and the steel strand. According to the principle of force interaction, the wedge anchorage system works through the interaction among the steel strand, wedges and the barrel. Once the steel strand is subjected to the tensile force P, the inner teeth of the wedges tightly grip the steel strands through the action of clamping force N, forming an effective contact area, meaning the signal from PZT 1 can be transmitted to the wedges through the contact surface. Since the clamping force N is the horizontal force of R, there is also an interaction force between the barrel and the wedges at the same time which causes the two rough elastic surfaces to become extruded. At the same moment, the steel strand and wedges can be regarded as a whole to interact with the barrel, which made PZT 3 receive signals from PZT 1 through the contact surface between the wedges and the barrel, thus realizing the energy transmission across contact interfaces for the wedge anchorage system. With the increase of the tension of the strand, the contact area between the anchors increases and the signal energy transmitted by PZT increases. Therefore, the tension state of prestressed steel strand can be monitored according to the transmitted signal value between different contact surfaces.

### 2.3. Detection Principle for Steel Strand Connection Based on Time Reversal

The time reversal method has good temporal and spatial focusing characteristics. It can be used to monitor the wedge anchorage system in the prestressed concrete structures.

In the experiment, the wedge anchorage system is utilized to connect the steel strands and the prestressed concrete structures. PZT 1, applied as an actuator, is attached to the top surface of the steel strand, and PZT 2, designed as a sensor, is pasted on the top of the wedges, as shown in Figures 2 and 5.

The signal *x*(*t*) input in PZT 1 is served as an excitation source, and the impulse response function is definite as *h*(*t*); the propagation signal y(t) obtained by PZT 2 can be shown as

(1) y(t)=x(t)⊗h(t)

where ⊗ represents the convolution operation.

The time reversal is done with the signal *y*(*t*) received by the PZT 2. The signal *y*(−*t*) obtained at PZT 2 according to time inversion can be expressed as

(2) y(−t)=x(−t)⊗h(−t)

Immediately, PZT 1 received the time reversed signal transmitted by PZT2. Therefore, the focused signal at PZT 1 that was obtained by time inversion can be expressed as

(3) $$y^{TR}(t)=y(-t)⊗h(t)=x(-t)⊗h(-t)⊗h(t)$$

where *h*(−*t*) and *h*(*t*) have the same transmission medium. According to the rules of convolution and correlation operations, $y^{TR}(t)$ can be expressed as

(4) $$y^{TR}(t)=x(-t)⊗\left[h(-t)*h(t)\right]$$

where ∗ presents the product operation.

A Gauss pulse is symmetric relative time, namely x(−t)=x(t). Thus, $y^{TR}(t)$ can be shown as

(5) $$y^{TR}(t)=x(t)⊗\left[h(-t)*h(t)\right]=x(t)\;{\left|h(t)\right|}^{2}$$

When *t* = 0, the focused signal at PZT 1 $y^{TR}(t)$ has the maximum value. Additionally, the peak value of time reversal focused signal is only related to the excitation signal and is not affected by the noise in the test environment.

The time reversal process for PZT 1 and PZT 3, and PZT 2 and PZT 3 is the same.

## 3. Experimental Setup

**Figure 3:**
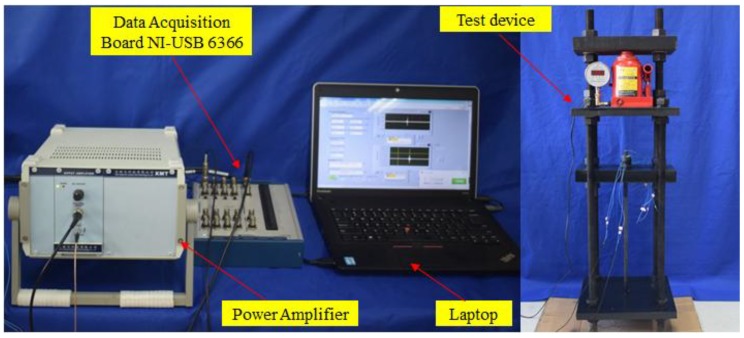
The experimental setup.

**Figure 4:**
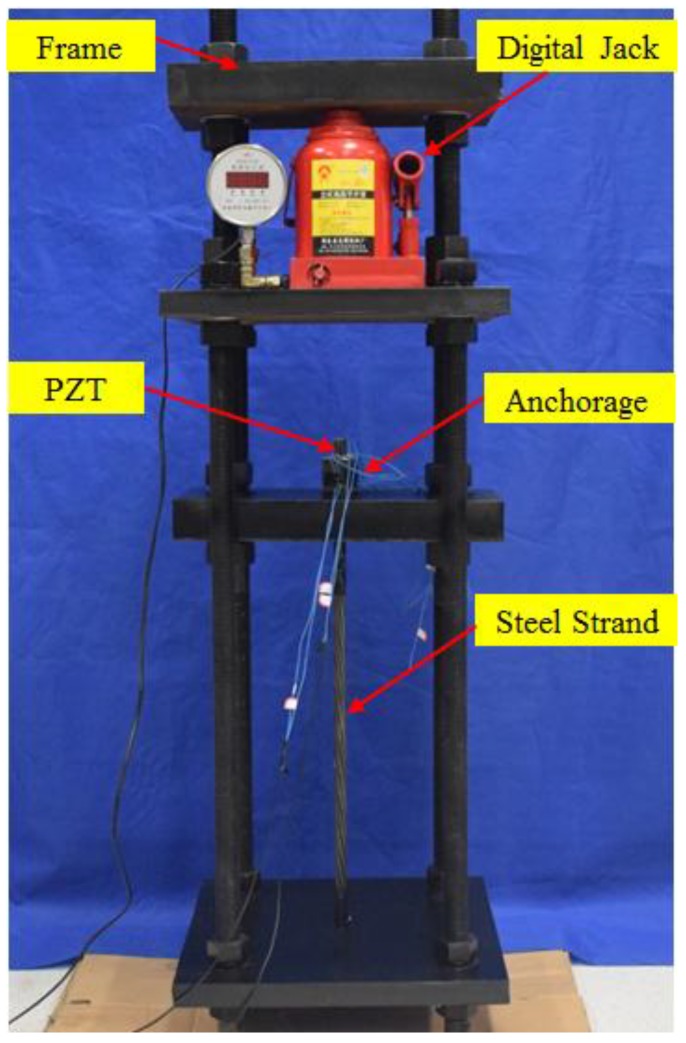
The test device.

**Figure 5:**
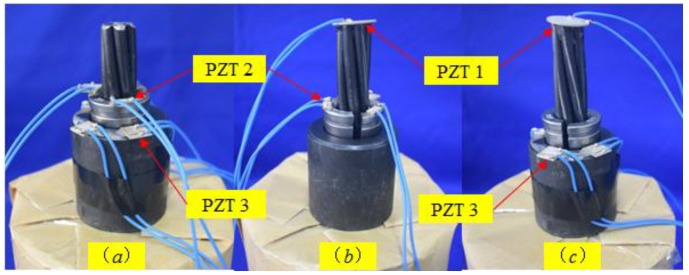
PZT locations for three contact surface monitoring methods of anchorage. (**a**): contact surface between the wedge and barrel; (**b**): contact surface between the steel strand and wedge; (**c**): contact surface between the steel strand and barrel.

The experimental setup of the wedge anchorage system monitoring mainly includes a steel strand and the wedge anchorage. The wedge anchorage consists of two wedges and a barrel. Three piezoelectric patches are bonded on the wedge anchorage system, an NI (National Instrument) data acquisition system and a hosting laptop computer, as shown in Figure 3. In the test, the steel strands were tensioned by the digital jack on the reaction frame under different tension levels, as shown in Figure 4. The wedge anchorage was gradually anchored as the tension of the steel strand increases. Three PZTs were respectively bonded on the surface of the steel strand, wedge and barrel. Once the steel strand was tensioned, two direct contact faces and an indirect contact face were formed through the interaction of the steel strand, wedges and barrel, as shown in Figure 5. There was a positive correlation between the tension of the steel strand and the contact areas. The peak value of the focused signal was changed as the tension of the digital jack was altered. According to the variation between the loading force of the digital jack and the peak value of the focused signal, the anchorage looseness of the steel strand can be monitored. In the practical application of bridge engineering, the peak value of the focused signal corresponding to the tension of the steel strand in the initial stage of the bridge can be used as the standard value. In the later operation of the bridge, the effective prestress loss of the steel strand can be monitored according to the attenuation of the peak value of the focused signal.

A Gauss pulse was first output via the D/A interface of the acquisition card and was then amplified by the amplifier before being sent to the actuator. Once the actuator was incentived, it generated a stress wave which was transmitted to a sensor through the wedge anchorage. The sensor converted the stress wave into an electrical signal which was obtained by the computer through the A/D interface of the NI data acquisition card. During the five independent experiments, the tension of the steel strands was divided into eight grades which were loaded step by step from 0 MPa to 20 MPa. After completing the data acquisition of one tension level, the steel strand was tensioned to the next tension level according to the loading sequence to collect the experimental data.

The experimental device includes the steel strands with the length of one meter and the diameter of 15.2 mm, a digital hydraulic jack with the weight of 20 t, two wedges and barrel anchorages. For the wedge anchorage that was used in the test device, its elastic modulus was 210 GPa. Taking theanchoring device that is now widely used in practical applications as an example, the clamping length of steel strand is 46mm and the inner diameter of the wedges is 14.5 mm. It can withstand up to 59% of the steel strand, while on the one hand, it must be ensured to hold the non-slip of the steel strand; on the other hand, the damage of the wedge to the steel strand must be reduced as far as possible so as to prevent the steel strand from breaking. After the steel strand is clamped through the wedges, the steel strand is gradually tensioned by the digital jacks on the counterforce frame. The values of different tensile forces indicate different anchoring conditions of the steel strand. The larger the tension forces of the digital jack, the greater the contact area between the steel strand and wedges. Nonconductive epoxy was utilized to bond three piezoelectric patches (PZT) to the surface of the steel strand, wedges and barrel, respectively.

The tension value conversion relationship is given in Table 1.

In order to verify the stability and feasibility of the time reversal method for monitoring the wedge anchorage system, anti-interference experiments were carried out during the experiment. In the course of the experiment, the external interference was produced by beating the anchorage system or the test device frame directly with a hammer. The anti-interference performance of the method was observed by comparing the peak values of the focused signals with and without the artificial interference.

## 4. Experimental Procedure, Results and Analyses

In the experiment, a pulse waveform was selected as the excitation signal to be transmitted out via the NI data acquisition card. Upon being sent out, the signal was magnified 10 times by the amplifier and was transmitted to the PZT 1, which was pasted on the steel strand. Once the PZT 1 was motived, the signal propagated through the wedges to the PZT 2 and attached on the wedges. Then the stress wave obtained at the PZT 2 was transformed into an electrical signal and was stored on the computer through the NI data acquisition card. The center frequency of the acquired signal was given in sweeping to be 100 kHz and the duration was 0.05 s. Then, the obtained signals under various loading conditions were collected.

According to the structural form of the wedge anchorage system, the monitoring of the anchorage tightness state for the prestressed steel strand consists of three methods. The first tightness monitoring method is according to the contact area between the steel strand and the wedges under different loading state. The second anchorage state monitoring method is according to the contact area between the steel strand and the barrel. The third method is depending on the contact surface between the wedges and the barrel. Prestressed steel strands are loaded with digital jacks under eight different tension levels. Finally, the focus value of the received signals based on the time reversal technique under different monitoring methods was compared analytically.

### 4.1. The Time-Reversal Test Between Steel Strand and Wedges

**Figure 6:**
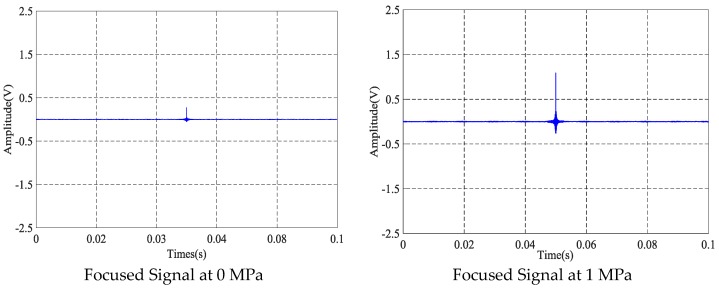

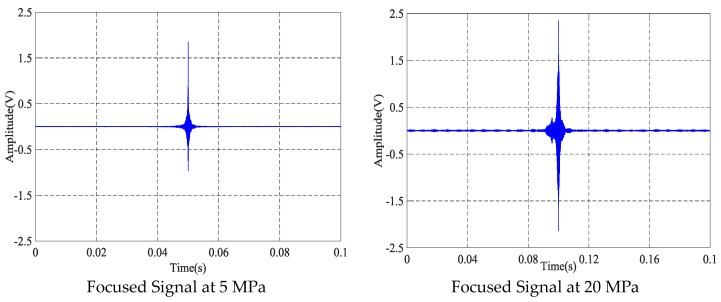
Focused signals based on the time reversal method at different tension levels.

**Figure 7:**
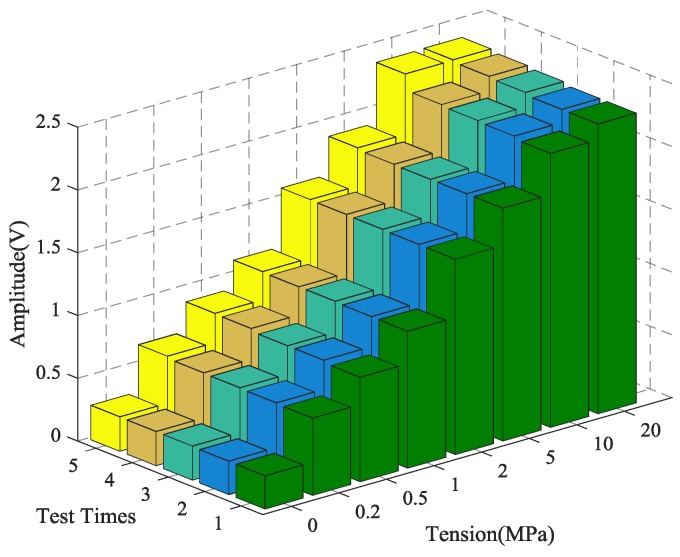
The magnitude of the focused signals based on time reversal for five repeated tests.

**Figure 8:**
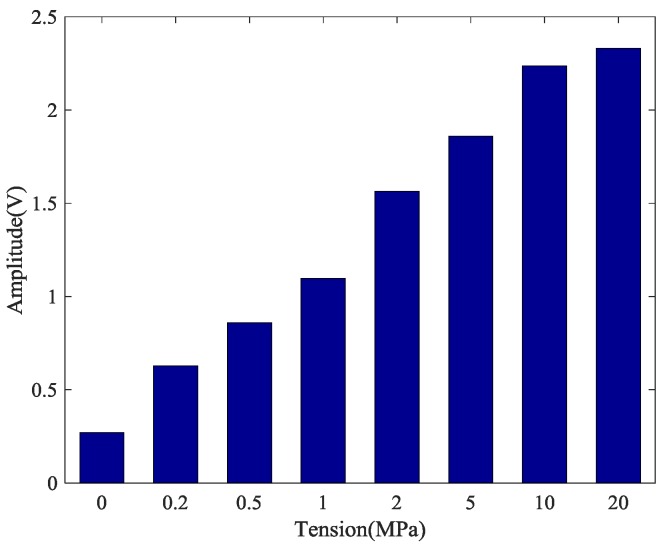
Mean of the magnitudes of the focused signals based on time reversal.

Using the time reversal technique, the tightness of the steel strand was monitored by selecting the focused value of the transmitted signal via the contact area between the steel strand and wedges. Due to space limitations, the peak value of focused signals under four tension states, including minimum, maximum and other two random groups of experimental loading conditions, was selected and is shown in Figure 6. It can be clearly seen that the maximum value of the collected signal increases evidently with the tension on the steel strand as the tension level increases from 0 MPa to 20 MPa, as shown in Figure 7 and Figure 8.

In order to verify the reliability and accuracy of the experimental results, five repeated tests were performed on the time reversal test between the steel strands and wedges. The steel strands with the same material characteristics were chosen for each test to ensure the independence of the experimental results. In addition, prior to every repeat test, the steel strand and wedge anchorage were replaced completely after the previous test.

The calculation results of the five tests are presented in Figure 7. The coefficient of variation (CV), shown as CV = *σ*/*μ*, is the absolute value of the standard deviation σ and mean value *μ*, which reflects the degree of dispersion of the test data. It can be observed in Figure 7 and Figure 8 that the monitoring of anchorage tightness for prestressed steel strand based on the time reversal method is repeatable and consistent. The coefficient variation of the five repeated test data in Table 2 indicates the feasibility of the test method, and the maximum CV is 0.02734821 for tension at 10 MPa and the minimum CV is 0.00348047 for tension at 2 MPa.

As shown in Table 2 and Figure 8, the data in the five experiments have deviations, however the peak value of the focused signals show a consistent trend with the increase of the tensile force of the steel strand. The average value of the five experimental data show that the peak value of the focused signals of the time reversal method is positively correlated with the tension of the steel strand, which can reflect the anchoring tightness of the steel strand. Finally, experimental data indicated that the steel strand tightness monitoring method between the steel strand and wedges has good repeatability and reliability, which suggests that it can be used in future practical applications.

### 4.2. The Time-Reversal Test Between Steel Strands and Barrel

**Figure 9:**
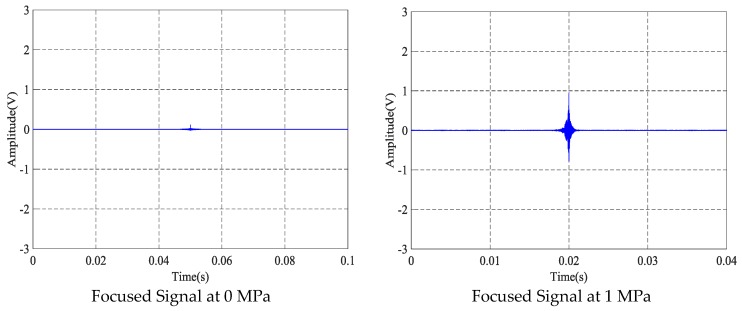

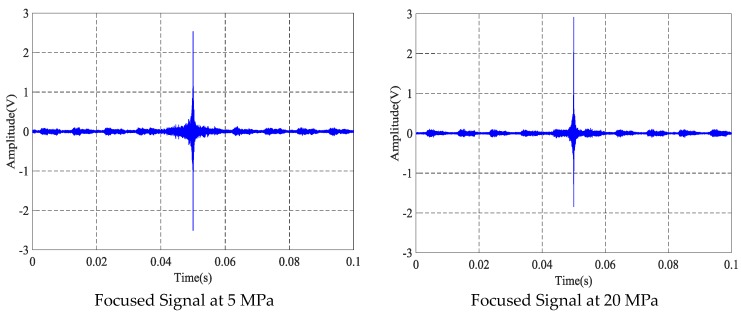
Focused signal based on the time reversal method at different tension levels.

Based on the time reversal method, the tightness of the steel strand was monitored by selecting the focused value of the transmitted signal via the contact area between the steel strand and barrel. Due to space limitations, the peak value of focused signals under four tension states, including minimum, maximum and other two random groups of experimental loading conditions, is selected and shown in Figure 9.

**Figure 10:**
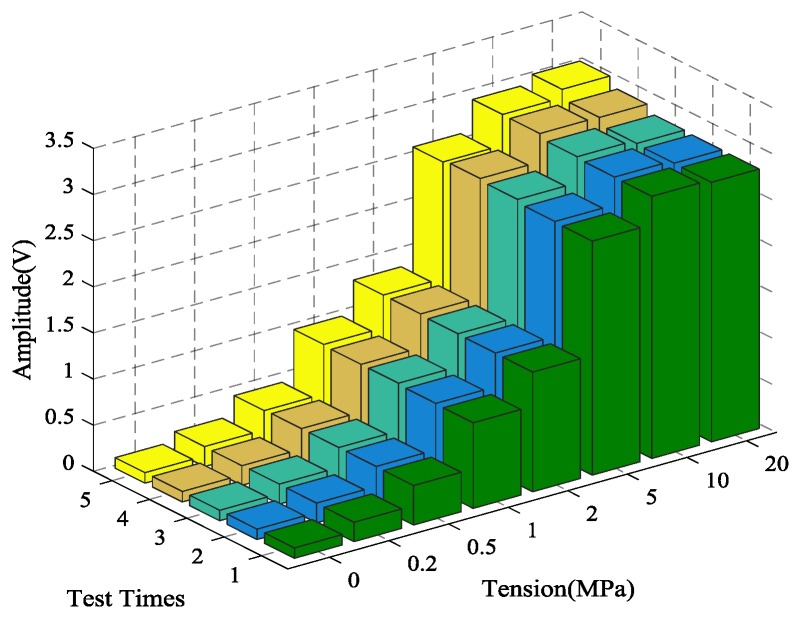
Focused signal based on times reversal for five repeated tests.

**Figure 11:**
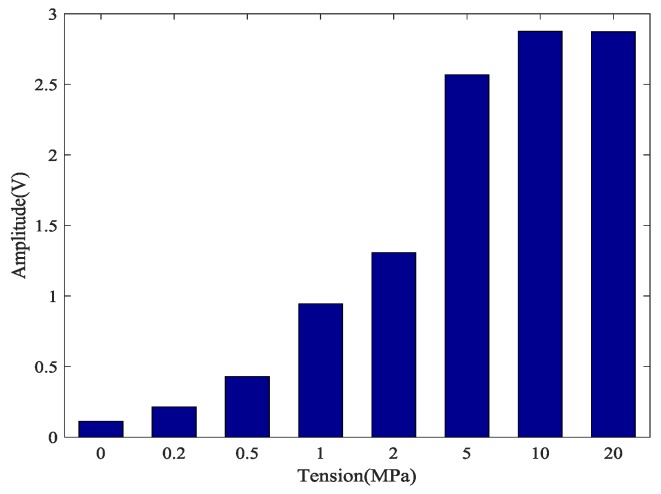
Mean of the magnitudes of the focused signals based on times reversal.

The repetitive test results are plotted in Figure 10 and Figure 11. From the results, it can be found that the monitoring of the tightness of the steel strand through the contact area between the steel strand and barrel is feasible and repeatable. In addition, the coefficient of variance calculated in Table 3 indicates that the experimental data have good consistency since the maximum CV is 0.04277936 at 0.5 MPa and the minimum CV is 0.00612221 at 0 MPa.

According to the principle of force interaction, the wedge anchorage system works through the interaction between the steel strand, wedges and the barrel. Once the steel strand was subjected to the tensile force, the inner teeth of the wedges tightly gripped the steel strands through the action of clamping force. Meanwhile, there is also an interaction force between the barrel and the wedges, which caused the two rough elastic surfaces to become extruded. Thus, the sensor can receive signals from the actuator across the contact surface, realizing the energy transmission across contact interfaces for the wedge anchorage system.

The results of five repeated experiments also show that the peak value of the focused signal between the steel strand and barrel is consistent with the trend of the tension of the steel strand. The anchor tightness of the steel strand can be monitored based on the peak value of the focus signal.

### 4.3. The Time-Reversal Test Between Wedges and Barrel

**Figure 12:**
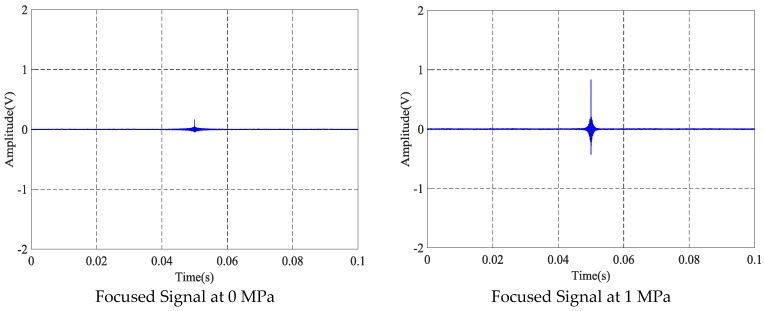

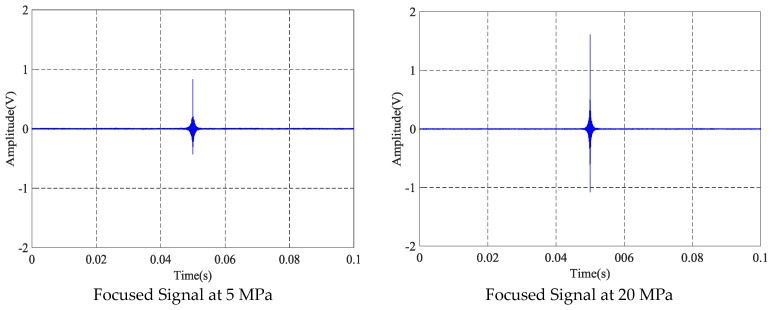
Focused signals based on the time reversal method at different tension levels.

Based on the time reversal method, the tightness of the steel strand was monitored by selecting the focused value of the transmitted signal via the contact area between the wedges and barrel. Due to space limitations, the peak value of focused signals under four tension states, including minimum, maximum and other two random groups of experimental loading conditions, is selected and shown in Figure 12.

**Figure 13:**
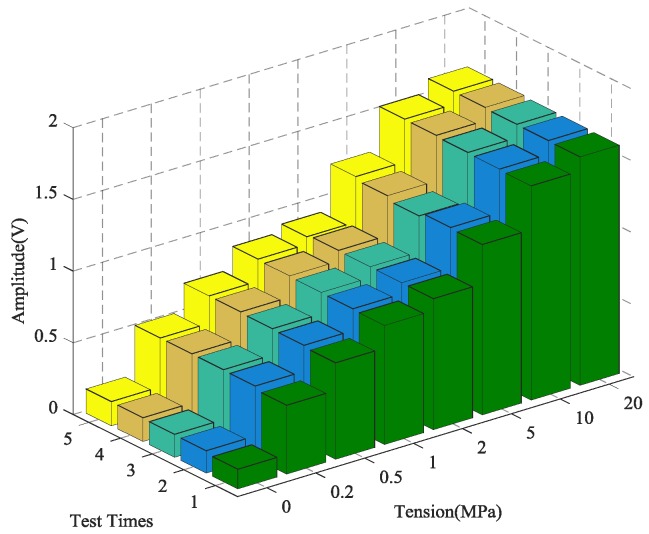
Magnitudes of focused signals based on times reversal for five repeated tests.

**Figure 14:**
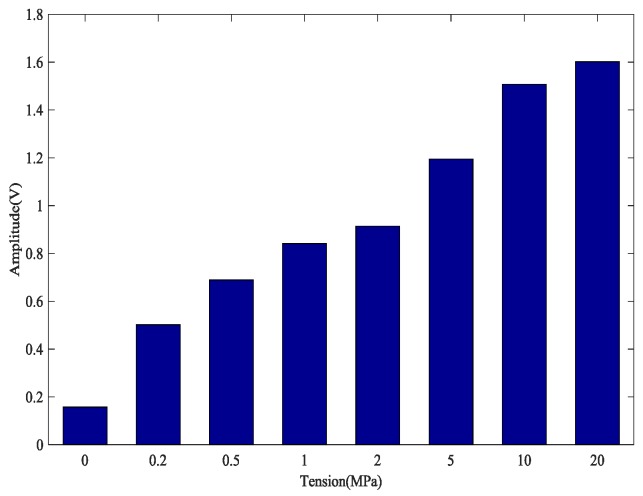
Mean of the focused signals based on times reversal.

From Figure 13 and Figure 14, it can be observed that the results of the five repeated experiments based on the contact area between wedges and barrel have good repeatability and consistency. The coefficient of variation in Table 4 further supports the above conclusions, since the maximum CV is 0.07086028 for the 0 MPa tension case and the minimum CV is 0.0053849 for the 2 MPa tension case. Therefore, this experimental method can be used to monitor the tension of the steel strands under the operating condition.

**Figure 15:**
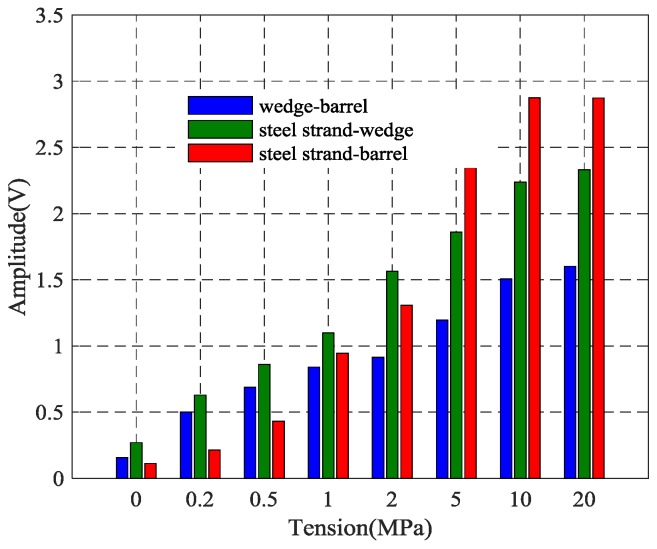
Three kinds of interface focused signal based on the time reversal method.

Focused values of the received signals from the three different contact surfaces of the prestressed steel strand anchorage system were compared by the time reversal method, as shown in Figure 15. The results indicated that the focused value of the received signal based on the time reversal technique through three different contact surfaces can reflect the tightness of the prestressed steel strand. The peak value of the retrieved signal has a positive correlation with the tensile force of the steel strand, namely, the peak value of the focused signal increases as the tension level of the steel strand increases. Moreover, the focus signal value between the wedge and barrel has the minimal variation, while the focus signal between the steel strand-wedges changes to become more stable and reliable. In the test, PZT 1 attached to the surface of the steel strand was selected as an actuator to generate an elastic stress wave and PZT 3 bonded on the barrel was utilized to detect the wave that propagates through the wedge anchorage system. Once the steel strand is tensioned and anchored, the steel strand, the wedges and barrel are tightly pressed together, and the signal from PZT 1 is transmitted to the barrel through the two wedges that closely surround, and the PZT 3 on the barrel will receive the signal from the steel strand and there is no loss in the signal transmission process. Therefore, the steel strand-barrel has the highest values, though two interfaces are involved.

Regarding the wedge anchorage system that consists of three parts that forms three different contact surfaces, each one has different signal transmission paths and different peak values of focused signals. However, the peak values of the focused signals under three different kinds of contact surfaces are consistent with the variation of the tensile force of the steel strands which can be used to monitor the anchoring tightness of the steel strand during the operation of the bridge.

### 4.4. Anti-Disturbance Ability Results and Analyses

**Figure 16:**
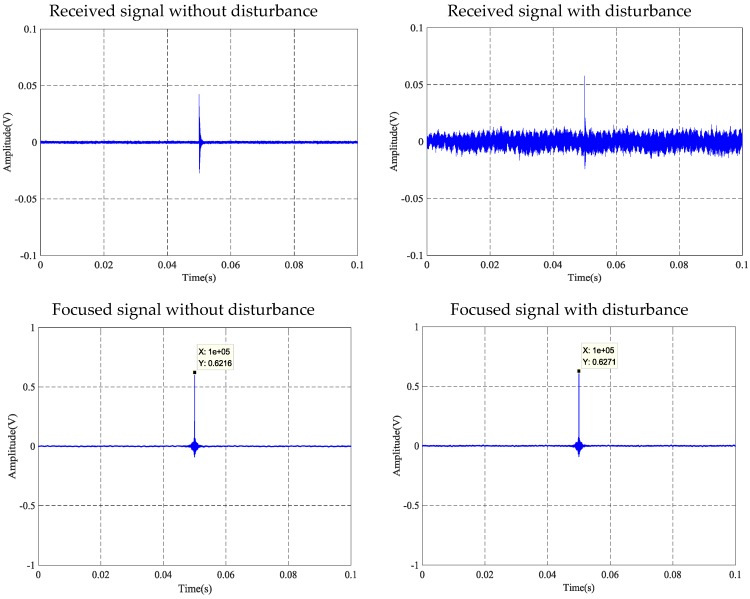
Anti-disturbance ability test at 0.2 MPa tension level of steel strand and wedges.

**Figure 17:**
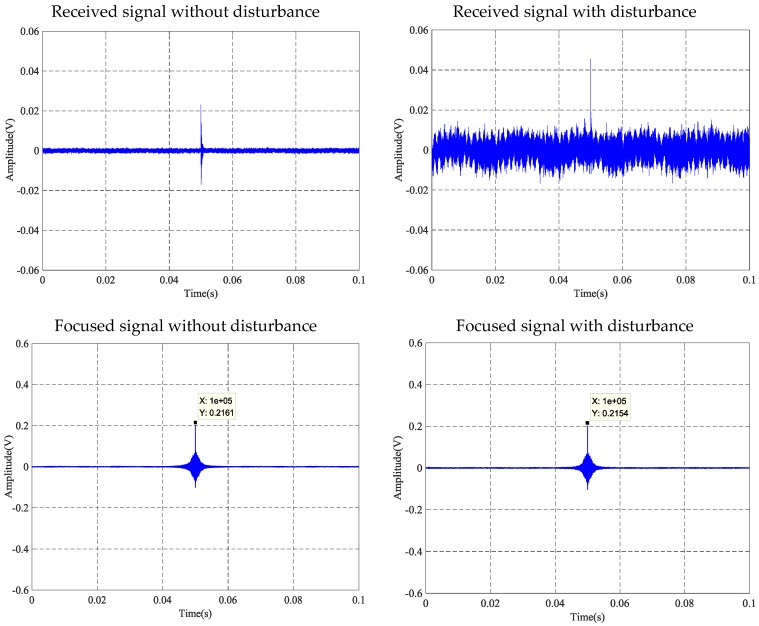
Anti-disturbance ability test at 0.2 MPa tension level of steel strand and barrel.

**Figure 18:**
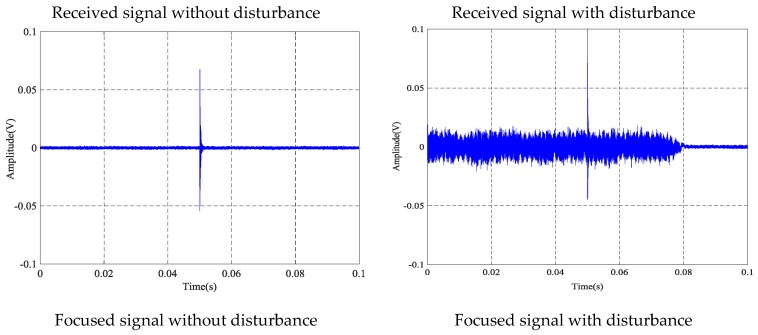

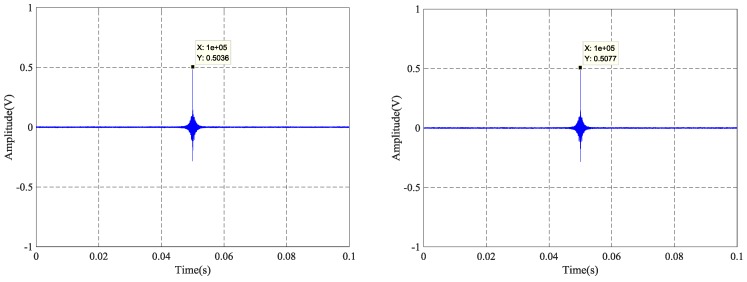
Anti-disturbance ability test at 0.2 MPa tension level of wedges and barrel.

Owing to space restrictions, only the tension of the steel strand at 0.2 MPa is presented and discussed in this paper. Results for other conditions share similar behavior. Time reversal signals affected by artificial interference of three contact surfaces at the tension level of 0.2 MPa are presented in Figure 16, Figure 17 and Figure 18. It can be viewed in three experimental tests that the received signal is substantially covered by the interfering signal, while the focused signal is almost unaffected by the interfering signal. The interference test of three kinds of contact surfaces shows that the anchoring of steel strands based on the time inversion method has superior anti-interference ability.

Figure 16 presents the results of the anti-interference test utilizing the contact surface between the strand and wedges. Compared with the interference effects between the received signal and the focused signal, the peak value of the focused signal for the strand tension at 0.2 MPa was 0.6271 V, which is less than the maximum value of the focus signal for 0.6491 V in the same tension state in Table 2. Thus, it can be judged as a normal operating state. At the same time, under the condition that the tensile force of the steel strand is 0.2 MPa, the peak value of the disturbed focus signal is consistent with the peak of the undisturbed focus signal. Anti-interference test results of the contact surface between the strand and wedges show that the TR (time reversal) method has good anti-interference ability.

Figure 17 presents the results of the anti-interference test utilizing the contact surface between the strand and barrel. Compared with the interference effects between the received signal and the focused signal, the peak value of the focused signal for the strand tension at 0.2 MPa is 0.2154 V, which is less than the maximum value of the focus signal for 0.216627 V in the same tension state in Table 3. Thus, it can be judged as a normal operating state. At the same time, under the condition that the tensile force of the steel strand is 0.2 MPa, the peak value of the disturbed focus signal is consistent with the peak of the undisturbed focus signal. Anti-interference test results of the contact surface between the strand and barrel show that the TR method has good anti-interference ability.

Figure 18 presents the results of the anti-interference test utilizing the contact surface between the wedges and barrel. Compared with the interference effects between the received signal and the focused signal, the peak value of the focused signal for the strand tension at 0.2 MPa is 0.5077 V, which is less than the maximum value of the focus signal for 0.508538 V in the same tension state in Table 4. Thus, it can be judged as a normal operating state. At the same time, under the condition that the tensile force of the steel strand is 0.2 MPa, the peak value of the disturbed focus signal is consistent with the peak of the undisturbed focus signal. Anti-interference test results of the contact surface between the wedges and barrel show that the TR method has good anti-interference ability.

## 5. Conclusions

The PZT based time reversal method that was presented in this paper can effectively monitor the anchoring tightness of prestressed steel strands under operational conditions. The increase in tension on the steel strand supplied by the digital jack leads to an increasing contact area among the steel strand, wedges and barrel in the wedge anchorage system, and contributes to the transmission of transmitted signals. Statistical experimental data show that the focused signal value that was calculated by the time reversal method decrease with the loosening of steel strand anchorage, which can reflect the attenuation of the tensile force of the prestressed steel strand. Furthermore, five repeated tests proved that the proposed test method has satisfactory feasibility and reliability. The test results verify the feasibility of the new method. Meanwhile, the test verified that the signal can be transmitted across the contact surface. The anti-disturbance experiment results show that the time reversal technique has good anti-disturbance performance and can guarantee the stability of the experimental results in a complex construction environment. In conclusion, all three experimental results indicate that the TR method can be utilized to monitor the looseness of effective prestress for prestressed steel strands during operation. The main purpose of this study is to verify the feasibility of the new method, and more in-depth research will be done in the future, including the development of wireless data acquisition systems and related product equipment. The PZT based time reversal method that was proposed in this paper provides the opportunity to be applied in health monitoring of other long-span prestressed structures with a wedge anchorage system, which has wide application prospects and practical value.

## Figures and Tables

**Table 1:** The relevant tension level of the hydraulic jack.

MPa	0	0.2	0.5	1	2	5	10	20
kN	0	0.276	0.69	1.38	2.76	6.9	13.8	27.6

**Table 2:** Five test’s results.

Tension/MPa	Tension/kN	Min/V	Max/V	*μ*/V	*σ*/*μ*/(10^−2^)
0	0	0.264558	0.274446	0.269632	1.419444
0.2	0.276	0.615164	0.6491	0.628855	1.826525
0.5	0.69	0.832016	0.881979	0.859474	2.088011
1	1.38	1.090825	1.106081	1.097808	0.506306
2	2.76	1.556926	1.571639	1.563922	0.348047
5	6.9	1.854132	1.875389	1.859441	0.439823
10	13.8	2.182169	1.875389	2.236725	2.734821
20	27.6	2.308493	2.359761	2.330045	0.844448

**Table 3:** Five test’s results.

Tension/MPa	Tension/kN	Min/V	Max/V	*μ*/V	*σ*/*μ*/(10^−2^)
0	0	0.11208	0.113996	0.112921	0.612221
0.2	0.276	0.209888	0.216627	0.214014	1.195999
0.5	0.69	0.386678	0.434445	0.422541	4.277936
1	1.38	0.927317	0.962595	0.94467	1.329268
2	2.76	1.296668	1.317607	1.307192	0.651326
5	6.9	2.535705	2.604062	2.569132	0.979111
10	13.8	2.840649	2.914029	2.875087	1.093778
20	27.6	2.812053	3.006242	2.873273	2.597293

**Table 4:** Five test’s results.

Tension/MPa	Tension/kN	Min/V	Max/V	*μ*/V	*σ*/*μ*/(10^−2^)
0	0	0.136850	0.168320	0.157398	7.086028
0.2	0.276	0.479997	0.508538	0.501667	2.189873
0.5	0.69	0.672501	0.698786	0.688785	1.399519
1	1.38	0.830159	0.852215	0.840823	0.895999
2	2.76	0.905172	0.920107	0.914026	0.53849
5	6.9	1.167486	1.221231	1.194364	1.452056
10	13.8	1.494013	1.519024	1.507248	0.616488
20	27.6	1.592577	1.610284	1.601367	0.413511
